# Effects of small *Hsp *genes on developmental stability and microenvironmental canalization

**DOI:** 10.1186/1471-2148-10-284

**Published:** 2010-09-16

**Authors:** Kazuo H Takahashi, Lea Rako, Toshiyuki Takano-Shimizu, Ary A Hoffmann, Siu F Lee

**Affiliations:** 1 Centre for Environmental Stress and Adaptation Research, Department of Genetics, Bio21 Institute, The University of Melbourne, Parkville, Melbourne 3010, Australia; 2 Department of Population Genetics, National Institute of Genetics, Mishima, Shizuoka-ken 411-8540, Japan; 3 Research Core for Interdisciplinary Sciences, Okayama University, Tsushimanaka 3-1-1, Kita-ku, Okayama City, Japan

## Abstract

**Background:**

Progression of development has to be insulated from the damaging impacts of environmental and genetic perturbations to produce highly predictable phenotypes. Molecular chaperones, such as the heat shock proteins (HSPs), are known to buffer various environmental stresses, and are deeply involved in protein homeostasis. These characteristics of HSPs imply that they might affect developmental buffering and canalization.

**Results:**

We examined the role of nine *Hsp *genes using the GAL4/UAS-RNAi system on phenotypic variation of various morphological traits in *Drosophila melanogaster*. The stability of bristle number, wing size and wing shape was characterized through fluctuating asymmetry (FA) and the coefficient of variation (CV), or among-individual variation. Progeny of the GAL4/*Hsp-*RNAi crosses tended to have reduced trait means for both wing size and wing shape. Transcriptional knockdown of *Hsp67Bc *and *Hsp22 *significantly increased FA of bristle number, while knockdown of *Hsp67Ba *significantly increased FA and among-individual variation of wing shape but only in males. Suppression of *Hsp67Bb *expression significantly increased among-individual variation of bristle number. The knockdown of gene expression was confirmed for *Hsp67Ba*, *Hsp67Bc*, *Hsp22*, and *Hsp67Bb*. Correlation between FA and CV or among-individual variation of each trait is weak and not significant except for the case of male wing shape.

**Conclusion:**

Four small *Hsp *genes (*Hsp22*, *Hsp67Ba*, *Hsp67Bb *and *Hsp67Bc*) showed involvement in the processes of morphogenesis and developmental stability. Due to possible different functions in terms of developmental buffering of these small *Hsp*s, phenotypic stability of an organism is probably maintained by multiple mechanisms triggered by different environmental and genetic stresses on different traits. This novel finding may lead to a better understanding of non-*Hsp90 *molecular mechanisms controlling variability in morphological traits.

## Background

Progression of development has to be insulated from the damaging impacts of environmental and genetic perturbations to produce highly predictable phenotypes. Waddington [1] suggested a conceptual mechanism called canalization that buffers developmental processes from environmental and genetic perturbations and therefore helps to produce constant phenotypes. Molecular chaperones, such as the heat shock proteins (HSPs), are known to buffer various environmental stresses, and are deeply involved in protein homeostasis [2]. Those characteristics of HSPs imply that they have potential to be candidates for developmental buffering and canalization.

Inhibition of *Hsp90*, one of the molecular chaperones, has been found to increase phenotypic diversity in various organisms such as *Drosophila*, *Arabidopsis*, and zebrafish [3-5]. It suggests that *Hsp90 *buffers developmental perturbations on morphological traits in these species. However, Milton *et al. *[6] and Debat *et al. *[7] found that a reduction of HSP90 activity did not affect phenotypic variation. Based on the mixed results, Debat *et al. *[7] suggested that *Hsp90 *is one of the multiple factors that participate in the developmental buffering of morphological traits rather than the only controlling factor.

*Hsp70 *is one of the most well-studied stress response genes, and is inducible by thermal and nutritional stresses and inbreeding in *D. melanogaster *[8-12]. Previous research suggests that *Hsp70 *contributes to stabilization of developmental processes, although results are somewhat inconsistent. Roberts and Feder [13] showed that increased copy number of *Hsp70 *significantly reduced developmental abnormalities, while Williams *et al. *[14] observed the opposite effect.

Other molecular chaperones such as *Hsp22*, *Hsp67*, *Hsp68*, and *Hsc70 *are also known to respond to environmental stresses [15-19]. It has been suggested that they contribute to thermotolerance [16], and some of them (*Hsp22*, *Hsp68*, and *Hsp70*) affect longevity [20]. Although details of the chaperone activity and the molecular mechanism of the *Hsp*-mediated stress resistance are largely unknown, it is possible that these genes may also affect developmental processes.

In *D. melanogaster*, expression of *Hsp *genes is not only rapidly up-regulated under environmental stresses, but is also regulated during normal development. In the absence of environmental stress, *Hsp68 *and *Hsp70 *mRNA are expressed at very low levels in most developmental stages, but they are at higher concentration in pupae [21]. *Hsp22*, *Hsp67Ba*, and *Hsp67Bc *also reach a maximum level of expression in the early pupal stage in the absence of environmental stress [21,22]. *Hsp67Bb *mRNA is detected during all the larval stages to early pupal stage in a tissue-specific manner [23]. The higher expression of the *Hsp*s in embryos and pupae coincides with major developmental events. At the embryonic stage the body plan is being formed, while key metamorphosis processes occur during the pupal stage. *Hsp *activities during these periods occur at a time when meticulous spatial expression patterns develop. Such temporal regulation of expression suggests that these *Hsp *genes might help stabilize developmental processes at critical times.

This study aims to investigate the potential role of a subset of non-*Hsp90 *heat shock protein genes in phenotypic variability using RNA interference. If *Hsp *genes are involved in buffering phenotypic variability, suppression of their expression might result in a decrease in developmental stability and canalization. Here, we define developmental stability as a set of mechanisms buffering developmental variation among replicated or symmetrical organs within a single organism, and microenvironmental canalization as an increase in the phenotypic variance of a morphological trait, following Debat and David [24]. We infer developmental stability by measuring fluctuating asymmetry (FA) and the coefficient of variation (CV) or among-individual variation of several bristle traits and wing traits. Using the actin-GAL4/UAS-*Hsp*-RNAi system, we found that transcriptional suppression of *Hsp22 *and *Hsp67Bc *affected FA of bristle traits. We also detected a significant positive correlation between FA and among-individual variation of wing shape under *Hsp67Ba *knockdown. Furthermore, *Hsp67Bb *may influence among-individual variation of bristle traits. Our findings suggest developmental roles for non-*Hsp90 *stress proteins in controlling the expression of morphological variability.

## Methods

We used RNAi knockdown to evaluate the effect of target *Hsp *genes. The crosses utilized a common GAL4 driver line in combination with one of the eight RNAi lines (transformant ID: 21806, 26416, 33207, 36641, 43632, 47145, 49795, 49796) developed by the Vienna Drosophila RNAi Center (VDRC). The target *Hsp *genes were *Hsp22*, *Hsp67Ba*, *Hsp67Bb*, *Hsp67Bc*, *Hsp68*, *Hsp70Ba*, *Hsp70Bb*, *Hsp70Bbb*, and *Hsp70Bc *(Table 1). All RNAi lines were constructed in an isogenic background (DSK001) as described in Dietzl *et al. *[25]. Although some of the RNAi strains (21806, 33207, 36641, 47145) have potential off-target genes, the specificity score, s_19_, was quite high (> 0.74) (Table 1), indicating highly specific knockdown of the target genes [25]. Females of a ubiquitous GAL4 driver strain, *y w; P{Act5C-*GAL4*}17bFO1/TM6B^Tb^*, were crossed to males of the RNAi lines to drive RNAi using GAL4-UAS system. The F_1 _offspring between GAL4 strain females and DSK001 males were used as a control.

**Table 1:** RNAi strains and their specificity score (s_19_), target and off-target genes.

Strain	s_19 _score	Target *Hsp *(s)	Predicted off-targets
21806	0.893	*Hsp67Ba*	*Pkcδ, CG14656, CG15725, CG15803, ASPP, CG30377, Samuel, shep, CG32541, Hs3st-A, CG33988, vir, Smr, bun, CG5697, CG5794, JIL-1, shn, Rgl, brk*
26416	1.000	*Hsp67Bc*	-
33207	0.806	*Hsp70Ba, Hsp70Bb, Hsp70Bbb, Hsp70Bc*	*Hsp68, Hsp70Aa, Hsp70Ab, Hsc70-2, Hsc70-1*
36641	0.747	*Hsp70Ba, Hsp70Bb, Hsp70Bbb, Hsp70Bc*	*Hsp70Aa, Hsp70Ab, Hsc70-1, Hsc70-2, CG14786*
43632	1.000	*Hsp22*	-
47145	0.997	*Hsp68*	*Trxr-2*
49795	1.000	*Hsp67Bb*	-
49796	1.000	*Hsp67Bb*	-

One hundred eggs were collected from each cross, and placed into a 42 ml glass vial with 10 ml of the fly medium. Fly food was made according to the Bloomington stock centre, where corn syrup was replaced with dextrose medium http://flystocks.bio.indiana.edu/Fly_Work/media-recipes/bloomfood.htm. Vials were then put into 27°C cabinets. Emerging adults were collected every day and preserved in 70% ethanol for morphological measurements. Five replicate vials were set up for each cross, and we measured three females and males from each vial.

### Morphological traits and shape analysis

**Figure 1:**
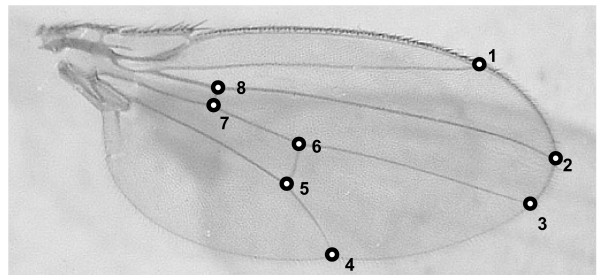
**Positions of eight landmarks used in this study**.

We measured both meristic and metric traits, as they may respond differently to a lack of microenvironmental canalization [26]. To evaluate the effect of *Hsp *knockdown on meristic traits, we scored five bristle traits - the number of sternopleurals (SP), scutellar (SC), thorax (TH), ocellar (OC), and orbital (OR) bristles on the right and left side of each fly. Principal component analysis (PCA) was performed to reduce the dimension of the bristle traits, and we used the first principal component (Bristle PC1) for further analysis. For metric traits, we measured an allometric (centroid size (CS)) and a non-allometric (wing shape (WS)) component of the wing trait, using the eight landmarks on the junctions between longitudinal veins and cross veins or wing margins (Figure 1). Firstly, we removed right and left wings of each individual and captured the wing images with a CCD camera attached to a microscope (WILD M3B (Heerbrugg, Switzerland)). The *x *and *y *coordinates of each landmark were obtained with the tpsDig2 program http://life.bio.sunysb.edu/morph/ and measured twice to evaluate the repeatability of the landmark acquisition. We then performed the Procrustes generalized least squares procedure [27-29] with the 'shapes' package of the statistical software R to obtain Procrustes coordinates. These were then used to perform relative warp analysis. Relative warp analysis is a principal component analysis based on covariance matrix where relative warps can be interpreted as principal component axes. Relative warps show the decompositions of shape changes ordered by their percentage of total variance explained. We used first relative warp (RW1: proportion of variance explained was 0.42) to characterize WS. We combined the data from both sexes for the above analyses.

To visualize the effect of RNAi on WS, we performed thin-plate spline analysis. Thin-plate splines indicate the differences in two configurations of landmarks as a continuous deformation using regression functions in which corresponding landmarks are matched between configurations to minimize the bending energy [30]. Bending energy is the energy required to bend an infinitely thin metal plate over one set of landmarks so that the height over each landmark is equal to the coordinates of the corresponding landmark in the other configuration [31]. We compared the mean wing shape of the GAL4/+ to each of the GAL4/UAS-RNAi crosses and then exaggerated their difference by 30-fold for graphical display.

For bristle traits and CS, FA was evaluated as |L-R|/(L+R)/2, where L indicates a trait value on the left side and R on the right side of the body. To evaluate FA of bristle traits collectively, we used a composite index of FA (CFA) proposed by Leung *et al*. (2000). To calculate this index (CFA2 in Leung *et al. *[32]), individual FA value was divided by the average FA of a given trait in the population of interest so that all traits contribute equally to CFA measure. The FA values were then summed across traits for each individual, creating a composite FA score for each individual. CFA2 is one of the best CFAs according to the simulation by Leung *et al*. (2000). We used all bristle traits to calculate CFA2. For WS, we used a univariate measure of FA devised by Klingenberg and Monteiro [33]. This measure is based on the idea of one-sample standard distance [34,35], and is equivalent to the one-sample version of the Mahalanobis distance [36], automatically providing a correction for directional asymmetry [33].

To evaluate the effect of RNAi knockdowns on microenvironmental canalization, we calculated among-individual variation of bristle traits, CS and WS. As for CS, variance and mean were calculated for each replicate vial to obtain the coefficient of variation (CV). As for bristle traits, we calculated the trace of the total covariance matrix for among-individual variation from all the components of PCA, and used it as an index of among-individual variation. As for WS, we performed the same analysis based on the relative warp, and used the trace as an index of among-individual variation. All these analyses were performed for females and males separately.

### Repeatability and measurement error of wing traits

To evaluate the measurement error in landmark acquisition based on two repeated measurements for each landmark, repeatability (*R*) was calculated for each landmark coordinate and CS [37]. The repeatability measure determines the proportion of variance due to variation between individuals where zero indicates that all variance is attributable to variance within individuals (100% measurement error) whilst one indicates all variance is found between individuals (0% measurement error).

To assess the relative amounts of directional asymmetry (DA), FA, and measurement error in wing shape variation, we employed Procrustes ANOVA [38] with degrees of freedom under the isotropic model [39]. In this analysis, we included individual, and side and their interaction terms, and added sums of squares across landmarks and coordinates, assuming equal and isotropic variation at each landmark.

### Analysis

To evaluate the effect of RNAi knockdown on FA and CV or among-individual variation, we performed Dunnett tests with bristle CFA, CS FA, WS FA, bristle among-individual variation, CS CV or WS among-individual variation as a dependent variable and RNAi strain as an independent variable. We applied Bonferroni correction to account for multiple Dunnett tests performed for each trait for each sex. The effect of RNAi knockdown on mean trait values was tested with the same analysis using Bristle PC1, CS or WS as dependent variables. We checked normality of the distributions using the Kolmogorov-Smirnov test. No significant deviations from normal distribution were detected for any measure.

### Quantitative real time PCR

We measured relative expression levels of *Hsp *genes in the early pupal stage of the GAL4/RNAi individuals and the control (GAL4/+) by performing quantitative real time PCR (RTPCR). Test larvae were produced by mating males from RNAi line with females of the GAL4 strain to drive RNAi, while control samples were obtained by crossing males of the control stain to females of the GAL4 strain. One hundred eggs were collected from each cross and placed on fly media as described earlier. Eggs were reared at 27°C, and larvae were sampled at a 70% of average pre-adult developmental time. Female and male pupae were not distinguished. We set up three biological replications and sampled 15 to 30 individuals for each replication.

Total RNA was extracted using TRIzol^® ^Reagent (Invitrogen Cat. No. 15596-026) for the GAL4/UAS-RNAi individuals using strains 26416, 33207, 36641, 43632, 47145, 49795, and 49796. Five *μ*g of total RNA was treated with 0.73 units of RQ1 RNase-Free DNase (Promega Cat. No. M6101) before converting to first strand cDNA using the SuperScript^® ^III First-Strand Synthesis SuperMix system (Invitrogen Cat. No. 18080-400). Oligo-dT was used to prime the reverse transcription. As for the GAL4/UAS-RNAi individuals using strain 21608, total RNA was extracted using the SV Total RNA Isolation System (Promega Cat. No. Z3100), and then converted to first strand cDNA using SuperScript III First Strand Synthesis System for RT-PCR (Cat. No. 18080-051) with Oligo-dT primer. cDNA was diluted 20 to 50 times in water.

Real time PCR was performed on the Roche LightCycler^® ^480. Five replications were set up per gene per cDNA sample. For the GAL4/UAS-RNAi individuals using strains 26416, 33207, 36641, 43632, 47145, 49795, 49796, we used 10 *μ*l reaction containing the following components: 1 *μ*l of cDNA, 1 *μ*l of Immolase 10× buffer, 0.4 *μ*l of MgCl_2 _(25 mM), 0.8 *μ*l of dNTP (2 mM), 4 *μ*l of forward and reverse primer mix (1 *μ*M each), 0.25 *μ*l of the LightCycler^® ^480 High Resolution Melting Master Mix (Roche Cat. No. 04909631001), 0.01 *μ*l of Immolase DNA polymerase (Bioline Cat. No. BIO-21047; 5 units per *μ*l), and 2.54 *μ*l of water. The thermocycling conditions were as follows: 95°C for 10 min, 50 cycles of 95°C for 5 sec, 58°C for 15 sec, and 72°C for 15 sec. Fluorescence signal was recorded at the end of each 72°C elongation phase. For GAL4/UAS-RNAi individuals using strain 21806, we used 20 *μ*l reaction that consisted of 2 *μ*l of cDNA, 4 *μ*l of forward and reverse primer mix (5 *μ*M), 10 *μ*l of LightCycler^® ^480 SYBR Green I Master (Cat. No. 4707516), and 4 *μ*l of water. The thermo cycling conditions were as follows: 95°C for 5 min, 45 cycles of 95°C for 10 sec, 60°C for 20 sec, and 72°C for 20 sec.

The Crossing point (Cp) estimates were acquired from the LightCycler^® ^480 using the Absolute Quantification Module. The conventional 2-ΔΔCt method was used to estimate relative gene expression. The mean Cp value was calculated for each cDNA sample based on five technical replicates. The relative expression of target gene was normalized with a housekeeping gene, ribosomal protein L11 (*RpL11*). The PCR primers for *RpL11 *for GAL4/21806 were described in Bogwitz *et al. *[40]. Mean normalized expression of the target gene was estimated based on three biological replications in each treatment. One-way ANOVA was performed to compare between two treatments (GAL4/UAS versus GAL4/+). The primers for real-time PCR are listed in Additional file: Appendix 1.

## Results

### Repeatability and measurement error

Repeatability of the acquisition of the individual landmark coordinates and CS was very high (R > 0.997 and 0.982 respectively), Procrustes ANOVA indicated that the contribution of measurement error to overall shape variation was small (Table 2), and the effect of FA was highly significant in all cases. The effect of DA was also significant except for males (Table 2).

**Table 2:** Procrustes ANOVA for the wing landmarks.

		**d.f**.	SS	MS	*F*	*P*
Female	Individual	1608	34929.160	21.722	3.250	< 0.0001
	Side	12	347.310	28.943	4.330	< 0.0001
	Individual × Side	1608	10748.460	6.684	5.330	< 0.0001
	Measurement error	3240	4063.020	1.254		
						
Male	Individual	1548	72199.030	46.640	2.753	< 0.0001
	Side	12	303.080	25.257	1.491	0.120
	Individual × Side	1548	26222.550	16.940	6.277	< 0.0001
	Measurement error	3120	8419.320	2.699		

Sums of squares (SS) and mean squares (MS) are in dimensionless units of Procrustes distance. The sums of squares are added over landmarks and coordinates, assuming that all landmarks have the same amount of isotropic variation.

### RNAi effect on trait means

**Figure 2:**
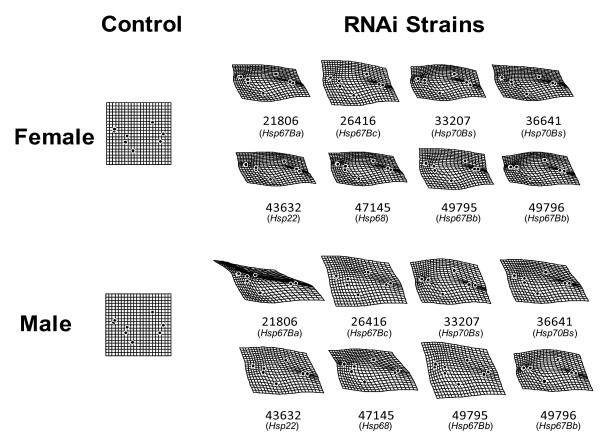
**Thin-plate spline (×30) between mean shape of GAL4/+ and mean shape of each of GAL4/UAS**.

The effects of the actin-GAL4/UAS-RNAi crosses in most cases were sex and trait dependent (Table 3). We observed a higher number of significant effects on bristle traits in males. The GAL4/26416 (targeting *Hsp67Bc*) was the only cross that had a significant effect on Bristle PC1 in females, whereas in males all crosses except for GAL4/21806 (targeting *Hsp67Ba*) and GAL4/49795 (targeting *Hsp68*) significantly reduced traits means. In general, the actin-GAL4/UAS-RNAi crosses reduced the trait mean of CS, and changed WS in a certain direction (Table 3). Six crosses reduced trait means for WS in females, and in males only four crosses (GAL4/21806, GAL4/26416, GAL4/36641 and GAL4/47145) had a significant effect. Thin-plate spline analysis showed that the degree of deflection in landmark configuration was strongest in GAL4/21806 males which also showed the strongest effect on mean WS (Table 3, Figure 2). The degree of deflection in landmark configuration in general seemed stronger in female (Figure 2), and more significant shape change was detected in female (Table 3).

**Table 3:** Mean score of Bristle PC1(OR), centroid size (CS), and wing shape (WS) of RNAi driven offspring from crosses between GAL4 and RNAi strains with standard deviation in parenthesis.

**Strain**	**Female**			**Male**		
	
	**Bristle PC1**	**CS**	**WS**	**Bristle PC1**	**CS**	**WS**
						
DSK001	2.483 (0.229)-	1.683 (0.012)-	6.985 (1.554)-	2.717 (0.532)-	1.490 (0.012)-	5.090 (0.564)-
21806 (*Hsp67Ba *)	2.573 (0.460)	1.600 (0.015)***	2.764 (1.432)**	2.168 (0.529)	1.361 (0.058)***	-11.158 (11.916)***
26416 (*Hsp67Bc *)	1.230 (0.331)**	1.652 (0.012)*	2.640 (1.139)**	0.969 (0.285)***	1.447 (0.008)	-4.074 (1.257)*
33207 (*Hsp70B *s)	2.227 (0.540)	1.653 (0.016)*	3.569 (1.199)*	1.449 (0.471)**	1.433 (0.012)*	-1.803 (1.359)
36641 (*Hsp70B *s)	2.575 (0.246)	1.684 (0.007)	2.979 (1.088)*	1.897 (0.190)	1.463 (0.015)	-4.969 (3.520)*
43632 (*Hsp22 *)	2.672 (0.377)	1.664 (0.019)	4.196 (1.904)	1.616 (0.526)**	1.434 (0.012)*	-3.209 (1.757)
47145 (*Hsp68 *)	2.499 (0.280)	1.659 (0.021)	2.783 (1.347)**	1.602 (0.546)**	1.400 (0.022)***	-5.483 (2.380)*
49795 (*Hsp67Bb *)	2.775 (0.766)	1.671 (0.007)	5.922 (1.761)	1.792 (0.403)	1.438(0.012)*	-2.727 (2.123)
49796 (*Hsp67Bb *)	2.702 (0.496)	1.677 (0.009)	1.724 (1.628)***	1.362 (0.385)***	1.457 (0.024)	-0.090(2.308)

*P < 0.05, **P < 0.01, ***P < 0.001 after Dunnett test and Bonferroni correction.

The phenotypic effect of each cross seemed to be trait specific except for GAL4/26416 (targeting *Hsp67Bc*) in females and GAL4/47145 (targeting *Hsp68*) in males. For example, the GAL4/21086 (targeting *Hsp67Ba*) affected CS and WS but there was no significant effect on bristle PC1. The GAL4/36641 (targeting *Hsp68*) also affected WS but not CS or bristle PC1 in either sex.

### RNAi effect on phenotypic variation and expression analysis

**Figure 3:**
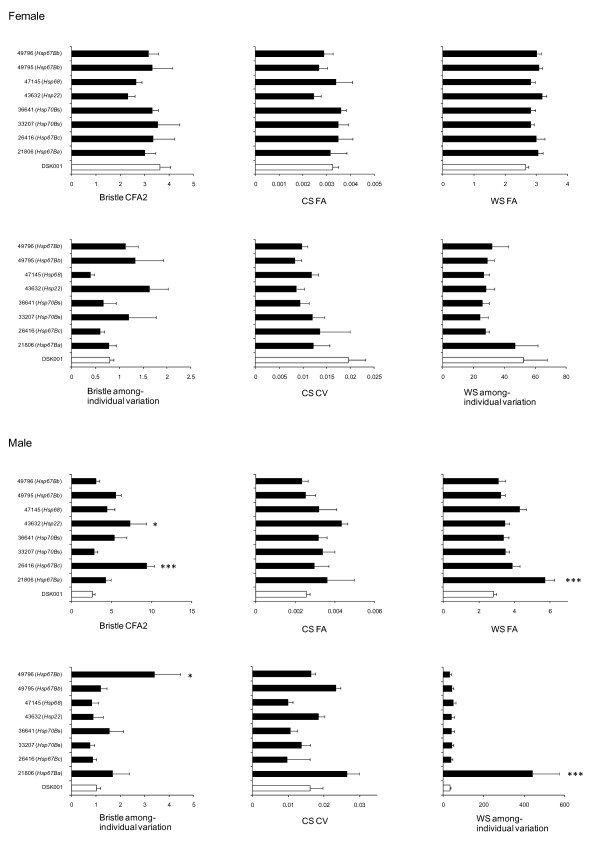
**Mean bristle composite FA2 (Bristle CFA2), Censtroid size FA (CS FA), Wing shape FA (WS FA), bristle among-individual variation (Bristle among-individual variation), Centroid size CV (CS CV) and Wing shape among-individual variation (WS among-individual variation) for female and male of GAL4/UAS and GAL4/+ individuals**. Open bars are for GAL4/+ control and closed bars are for the GAL4/UAS. Error bars represent standard errors. Asterisks indicate significant differences between each GAL4/UAS and the GAL4/+ control by multiple comparisons using Bonferroni method (*: *P *< 0.05, **: *P *< 0.01, ***: *P *< 0.001).

**Figure 4:**
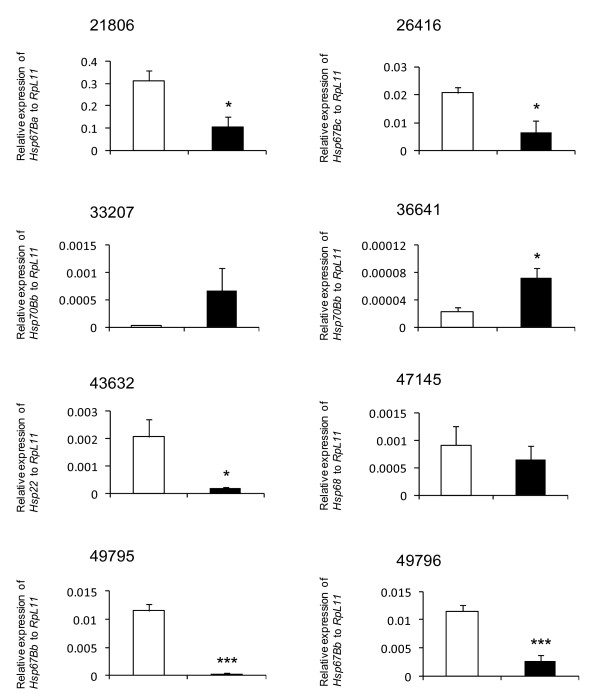
**Level of *Hsp22*, *Hsp67Ba*, *Hsp67Bb*, *Hsp67Bc*, *Hsp68*, and *Hsp70Bb *mRNA relative to *RpL11 *for GAL4/UAS (closed bars) and GAL4/+ (open bars) determined by RTPCR**. Error bars represent standard errors. Asterisks indicate significant difference between each GAL4/UAS and the GAL4/+ control heterozygote by ANOVA (*: *P *< 0.05, **: *P *< 0.01, ***: *P *< 0.001).

All the significant effects of RNAi on phenotypic variation were detected only in males. CS did not show significant change in phenotypic variation in any case in this study (Figure 3). RNAi knockdown of *Hsp67Bc *in GAL4/26416 and *Hsp22 *in GAL4/43632 significantly increased Bristle CFA2 (Figure 3). Expression levels of *Hsp67Bc *and *Hsp22 *were significantly knocked down (about 70% and 90% reduction respectively compared to the GAL4/+ control) (Figure 4). However, these changes did not alter FA or among-individual variation of wing traits, among-individual variation of bristle. On the other hand, while a significant reduction in the expression of *Hsp67Ba *in GAL4/21806 (66% reduction) did not affect Bristle CFA2 nor Bristle among-individual variation, it significantly affected WS FA and WS among-individual variation (Figure 3). A significant reduction of *Hsp67Bb *expression was achieved in both GAL4/49795 (96% reduction) and GAL4/49796 (77% reduction) but we detected a significant effect on bristle among-individual variation only in GAL4/49796 (Figure 4).

We were unable to detect a significant change in the expression of *Hsp70Bb *in GAL4/33207 and *Hsp68 *in GAL4/47145 (Figure 4), and we found no significant effect on FA or CV of any traits in either cross (Figure 3). We therefore could not test for an effect of *Hsp *knockdown on phenotypic variation for these genes. We observed a significant upregulation of *Hsp70Bb *in the GAL4/36641 cross (more than 300% increase), indicating that RNAi promoted instead of suppressed transcription. This overexpression of *Hsp70Bb *did not have a significant effect on phenotypic variation of any traits (Figure 3). The correlation between FA and CV of each trait is generally weak and not significant except in the case of WS in males (Table 4).

**Table 4:** Correlation coefficient of the pair of FA and CV of centroid size (CS), and among-individual variation of bristle and wing shape (WS)

	Female	Male
Bristle CFA2 vs. Bristle among-individual variation	0.081	-0.032
CS FA vs. CS CV	0.102	-0.051
WS FA vs. WS among-individual variation	-0.067	0.606***

***P < 0.001 after Bonferroni correction.

## Discussion

In this study, we tested whether dampened *Hsp *expression affects developmental stability and canalization in *D. melanogaster*. We attempted to knock down individual *Hsp *genes using the GAL4/UAS-RNAi system and measure various aspects of wing and bristle morphology. Evidence of gene suppression and phenotypic consequences on developmental stability and canalization were obtained for *Hsp22, Hsp67Ba, Hsp67Bb*, and *Hsp67Bc *knockdown, whereas results from RNAi targeting of *Hsp68 *and *Hsp70Bb *were inconclusive. We observed that the phenotypic outcomes on developmental stability and canalization of the four small *Hsp *knockdown were sex- and trait-dependent. It is known that the random integration of P-element-UAS constructs in the genome causes variability in the expression level of double-stranded RNA [41], and this might affect RNAi efficiency in this study. However, given that all RNAi strains used in the current study have an isogenic background except for the P-element insertion, the phenotypic effects are likely to be caused by reduced expression of the target and/or off-target genes.

Our results indicate that *Hsp22 *affects bristle but not wing shape asymmetry. *Drosophila *bristles or macrochaetae are important peripheral sensory organs; their spatial organization is likely to be a canalized trait. In a genetic modifier screen, Pena-Rangel *et al. *[42] found that alteration of *Hsp22 *expression (line EP(3)3247) could suppress the *pannier *(*pnr*) mutant phenotype, suggesting a genetic interaction between *Hsp22 *and mutant expression. PNR is a zinc finger transcription factor which activates *wingless *and the *achaete-scute *genes, which are crucial for bristle determination [43,44]. Together with other patterning regulators, PNR helps define the permissive (bristle mother cells) and restrictive regions for subsequent formation of the bristles [43]. The function of *Hsp22 *in buffering the phenotypic effect of a *pnr *mutation supports the idea that *Hsp22 *is involved in developmental stability of bristle traits. By examining the phenotypic effect of *pnr *mutant phenotype under overexpression or suppression of the expression of *Hsp22 *in a developmental stage specific manner, a possible link between *Hsp22 *and *pnr *could be investigated further.

The development of *Drosophila *bristles might parallel that of the nervous system. Held [45] suggested a connection between bristle patterning and embryonic neuroblast development [17]). Other small *Hsp*s such as *Hsp26 *and *Hsp27 *have been found through *in situ *hybridization to be expressed in embryonic central as well as peripheral nervous systems (BDGP database). Given the fact that RNAi knockdown of *Hsp22 *affected the mean bristle PC1 in this study, there may be co-localization of *Hsp22 *in the embryonic CNS tissues, and *Hsp22 *might function to buffer developmental perturbation in both tissues, but this remains to be tested.

Similar to *Hsp22*, knockdown of *Hsp67Bc *affects bristle trait asymmetry but not wing trait asymmetry. *Hsp22 *and *Hsp67Bc *are located at the chromosomal position of 67B on chromosome arm 3R, together with other small *Hsp*s, forming the 67B *Hsp *cluster. RNAi knockdown of either gene alone was sufficient to alter bristle phenotype. This suggests that these two genes have unique and indispensable roles in bristle development. At the peptide level, *Hsp22 *and *Hsp67Bc *share no obvious similarity in two regions that are suggested to affect chaperone activity [46]. At the transcriptional level, the tissue enrichment patterns of *Hsp67Bc *only partially overlap with those of *Hsp22 *(FLYATLAS http://www.flyatlas.org). Based on the dissimilarity in protein sequence, and non-overlapping spatial expression patterns, it is likely that *Hsp22 *and *Hsp67Bc *interact with different sets of protein clients, and contribute to bristle development through different mechanisms.

*Hsp67Ba *showed significant effects on both FA and among-individual variation of wing shape, suggesting that some developmental buffering mechanisms affect both within- and among-individual phenotypic variation. There was also a significant reduction of trait means in wing shape. However, there are at least 20 potential off-targets of this particular RNAi construct (VDRC construct ID 11237). It is therefore unclear whether the wing phenotypes we observed were due to successful suppression of *Hsp67Ba*, or collateral knockdown effects of these off-target genes. Future experiments should be directed to using an *Hsp67Ba *specific RNAi construct to assay for its causal knockdown phenotype. In fact, a new RNAi strain library, the KK library is available now at the Vienna *Drosophila *RNAi Center, in which each construct is targeted to the same position in the genome to reduce the number of off-target genes and positional effect. However coverage of the KK library is currently less than the RNAi strain library (GD library).

The RNAi lines 49795 and 49796 both target *Hsp67Bb *using the identical RNAi construct (VDRC construct ID 17696), and yet the phenotypic outcomes between these crosses were different. Bristle among-individual variation was significantly affected only in progeny derived from GAL4/49796. RTPCR results, however, indicated that *Hsp67Bb *was successfully suppressed in both crosses (Figure 4). One possible explanation for this inconsistent result is that there is a position effect of the construct, and the expression of *Hsp67Bb *may be reduced at different developmental stages in the RNAi lines. It is unclear at which pre-adult developmental stage(s) the mRNA knockdown exerts its effects on adult morphology. Future experiments could utilize stage-specific GAL4 driver lines to clarify the timing of the gene action.

The manner in which *sHsp*s tested in the present study buffer developmental variation is unknown. Rutherford *et al. *[47] suggested a hypothetical mechanism of *Hsp90 *developmental buffering, based on the idea of thresholds for the expression of phenotypes in response to continuously varying strengths of signaling through *Hsp90 *targeted pathways. In their hypothesis, when *Hsp90 *levels are decreased, signal transduction clients begin to lose activity, and the strength of target pathways becomes severely reduced. In specific genetic interactions between *Hsp90 *and signaling pathways, reduction of the signaling to the threshold for the expression of mutant phenotype reveals cryptic variation. Whether *sHsp*s affecting developmental buffering in this study have similar interactions with signaling pathways is unknown. The expression of *sHsp*s is regulated by the steroid molting hormone ecdysone and other enhancer elements [18], suggesting a possibility of their interaction with a number of signaling pathways. Recently, Specchia *et al. *[48] suggested a novel hypothesis that *Hsp90 *prevents phenotypic abnormalities by suppressing the mutagenic activity of transposons. They found that functional alteration of *Hsp90 *resulted in transposon activation and the induction of morphological mutants which indicated that *Hsp90 *mutation or inhibition can generate new genetic variation by transposon-mediated mutagenesis [48]. It is not known if *sHsp*s affect transposon activity.

Our data provide information on a long standing argument about whether there is a single or multiple developmental buffering mechanisms controlling developmental stability and canalization [49]. As suggested by some previous work on *Hsp90 *[6,50] and this study, multiple developmental buffering mechanisms may operate with trait specific effects. We found that overall the correlation between within- and among-individual phenotypic variations was very weak for most traits. This suggests that developmental stability and canalization are not always mediated by the same molecular machinery. Given that four *sHsp*s (*Hsp22*, *Hsp67Ba*, *Hsp67Bb*, and *Hsp67Bc*) showed gene-specific functions in terms of developmental buffering, phenotypic stability of an organism is probably maintained by multiple buffering mechanisms, activated by different environmental and/or genetic stresses for different traits.

## Conclusions

We identified four small *Hsp *genes (*Hsp22*, *Hsp67Bc*, *Hsp67Ba *and *Hsp67Bb*) that may influence developmental stability and/or canalization, possibly through multiple buffering mechanisms. Our findings provide new insights into the endogenous roles of these heat shock genes, and contribute to the understanding of the molecular mechanisms controlling variability in morphological traits. They also highlight that multiple genes can influence patterns of phenotypic variability.

## Authors' contributions

KHT designed the experiments. KHT, SFL and LR conducted the experiments. KHT analyzed and KHT, SFL and AAH interpreted the data and drafted the manuscript. All authors revised the manuscript critically for important intellectual content, participated in the discussions and approved its final form.
